# P-1065. Evaluating the Trends In Antimicrobial Prescriptions For Treatment Of Carbapenem Resistant Acinetobacter Baumannii Bacteremia And Clinical Outcome Differences In Polymyxin Based And Non Polymyxin Based Regimes

**DOI:** 10.1093/ofid/ofaf695.1260

**Published:** 2026-01-11

**Authors:** chaitanya Prabaharan, Merlin Moni, Dipu T Sathyapalan, preetha prasanna, Anandakrishnan Nandakumar, georg gutjahr, Ananth Ram K J

**Affiliations:** AMRITA INSTITUTE OF MEDICAL SCIENCES KOCHI,AMRITA VISHWA VIDYAPEETHAM, kochi, Kerala, India; Amrita Institute of Medical Sciences, Kochi, Kochi, Kerala, India; Professor, Department of Internal Medicine, Lead Division of Infectious diseases, Administrative chair, URUM, chairman HICC, Kochi, Kerala, India; AMRITA INSTITUTE OF MEDICAL SCIENCES KOCHI,AMRITA VISHWA VIDYAPEETHAM, kochi, Kerala, India; AMRITA SCHOOL OF PHYSICAL SCIENCES ,AMRITA VISHWA VIDYAPEETHAM, THRISSUR, Kerala, India; DEPARTMENT OF HEALTH SCIENCES AND RESEARCH,AMRITA VISHWA VIDYAPEETHAM, VIENNA, Wien, Austria; Clinical Pharmacist, Amrita Institute of Medical Sciences,AMRITA VISHWA VIDYAPEETHAM, Kochi, Kerala, India

## Abstract

**Background:**

Real-world data on outcomes of CRAB infections is scarce although incidence is higher in LMICs. Although currently IDSA recommends using Sulbactam-Durlobactam, due to limited availability in LMICs, Ampicillin-Sulbactam and Polymyxin-based combination therapies are commonly used. This retrospective single-centre study aimed to describe the trends in the antimicrobial regimens used for CRAB bacteremia and identify differences in clinical outcomes between Polymyxin-based and non-Polymyxin-based therapies.

**Figure:**
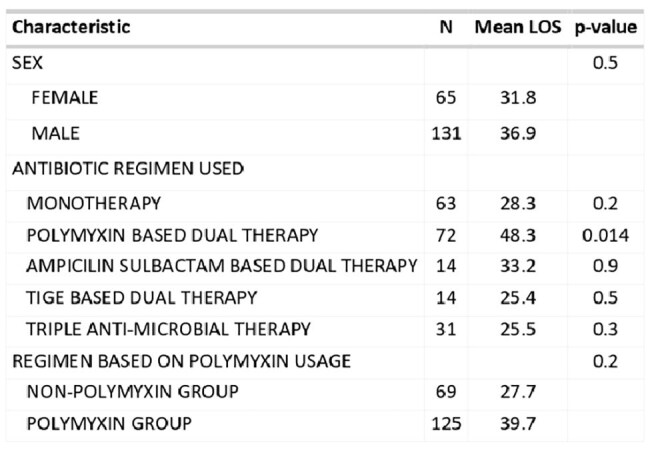

**Figure:**
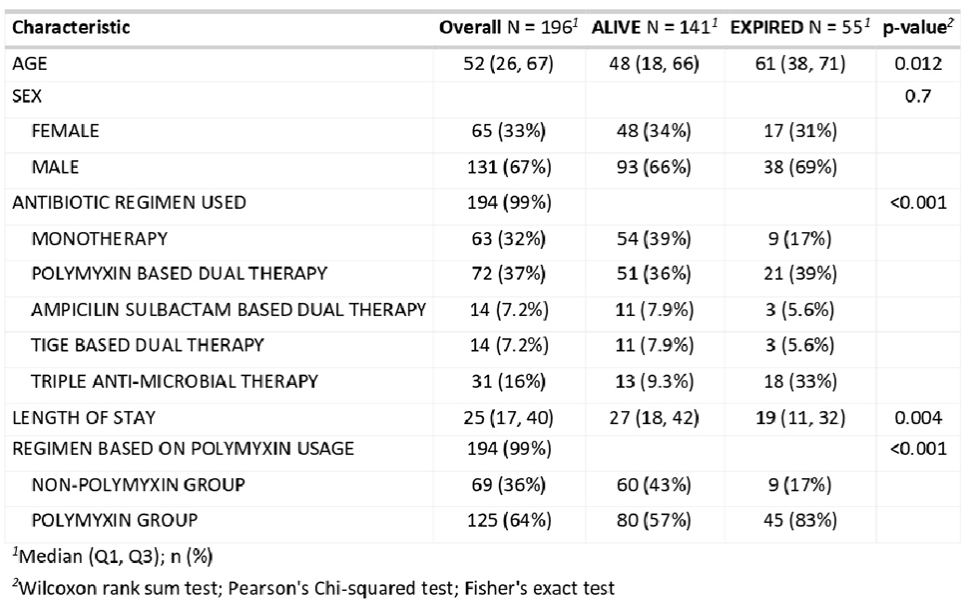

**Methods:**

Retrospective data on antimicrobial prescriptions, epidemiology, and clinical outcomes of patients with blood culture positivity for CRAB were captured through review of electronic medical records. Trends of antimicrobial prescriptions were identified and clinical outcomes of Polymyxin-based and non-Polymyxin-based regimens were compared

**Results:**

During the study period (January 2016–January 2025), 196 patients with CRAB bacteremia were identified. The median age of the cohort was 52 years (IQR 26–67), and 67% were male. In the majority of cases, bacteremia was primary. Among these, 125 patients (64%) were treated with Polymyxin-based regimens, while 69 patients (36%) received non-Polymyxin-based therapy, predominantly Ampicillin-Sulbactam-based combinations. The most commonly used agent for combination therapy was Polymyxin-based dual therapy (37%). The all-cause 28-day mortality of the cohort was 28%. Mortality in the Polymyxin-based and non-Polymyxin-based regimens was 36% and 13%, respectively (p< 0.01). The mortality of patients with CRAB bacteremia was 28%. Length of hospital stay was significantly shorter in patients receiving non-Polymyxin regimens (mean 27.7 days) compared to those on Polymyxins (mean 39.7 days), with a significant association between Polymyxin-based dual therapy and increased length of stay (p = 0.014).

**Conclusion:**

In this large single-centre cohort of CRAB bacteremia, Polymyxin-based regimens were more commonly used but were associated with higher mortality and prolonged hospital stay. Non-Polymyxin-based therapies, particularly Ampicillin-Sulbactam combinations, may offer a favorable alternative in settings with limited access to novel agents like Sulbactam-Durlobactam.

**Disclosures:**

All Authors: No reported disclosures

